# FloSeal® for Parenchymal Hemostasis in Robot-Assisted Partial Nephrectomy: Technical Description and a Report of Two Cases

**DOI:** 10.7759/cureus.103373

**Published:** 2026-02-10

**Authors:** Daisuke Igarashi, Go Kaneko, Suguru Shirotake, Masafumi Oyama

**Affiliations:** 1 Uro-Oncology, Saitama Medical University International Medical Center, Hidaka, JPN

**Keywords:** da vinci, da vinci 5, floseal, gelatin–thrombin matrix, hemostasis, partial nephrectomy, renal cell carcinoma, robot-assisted partial nephrectomy

## Abstract

Robot-assisted partial nephrectomy (RAPN) requires secure and efficient hemostasis at the resection margin to prevent bleeding-related complications while preserving renal function. Flowable gelatin-thrombin matrix sealants, such as FloSeal®, are useful adjuncts for hemostasis in partial nephrectomy; however, their use is limited in Japan. We report two cases of right-sided clinical T1a renal cell carcinoma treated with RAPN in which a gelatin-thrombin matrix sealant was applied. Standard tumor excision, meticulous coagulation, and inner-layer parenchymal renorrhaphy were successfully performed under renal artery clamping. A 14-Fr flexible feeding tube was then introduced through the assistant port to deliver an adequate amount of FloSeal® to the resection bed, after which the sealant layer was gently compressed with saline-moistened gauze. This procedure achieved complete parenchymal hemostasis after clamp release without the need for extensive cortical suturing. Both surgeries were completed without conversion and with minimal blood loss, negative surgical margins, uneventful postoperative courses, and preservation of early postoperative renal function. These two cases illustrate a practical technique for precise delivery of FloSeal® using a low-cost feeding tube during RAPN. This approach may serve as an option for standardized parenchymal hemostasis, particularly when the surgeon aims to minimize cortical suturing and warm ischemia time. Further studies are warranted to investigate the indications and long-term outcomes of this FloSeal®-assisted hemostatic strategy in nephron-sparing surgery.

## Introduction

Robot-assisted partial nephrectomy (RAPN) has rapidly gained widespread adoption and has become the standard treatment for small renal tumors because it enables precise tumor resection with excellent preservation of renal function. In partial nephrectomy (PN), after occlusion of the renal artery, the tumor is resected and the urinary tract is repaired when necessary. Reliable hemostasis at the resection margin is then achieved by various methods such as parenchymal suturing or the application of hemostatic agents. Because serious complications can result from bleeding at the resection margin, secure hemostasis is important. However, despite the central role of hemostasis in RAPN, detailed technical descriptions of how to integrate adjunctive flowable hemostatic agents into the procedure remain scarce, particularly in Japan. 

FloSeal® (Baxter Medical, Fremont, CA, USA) is a gelatin-thrombin matrix engineered to adhere even to irregular tissue surfaces and promote rapid coagulation. Its efficacy as a hemostatic agent during open and laparoscopic PN [1,2] and RAPN [3] has been reported. Previous studies have reported that local application of FloSeal® to the tumor bed after clamp release helps control residual arterial bleeding and parenchymal oozing, thereby reducing the need for extensive cortical suturing that may adversely affect postoperative renal function [4,5]. This may lower the risk of suture-related vascular complications such as arteriovenous fistulas and pseudoaneurysms [6]. In practical terms, this approach differs from conventional cortical renorrhaphy strategies that rely on multiple outer-layer compression sutures and sliding clips by instead combining limited inner-layer suturing with targeted application of a flowable hemostatic matrix. 

Despite these advantages, FloSeal® has not become widely used in PN in Japan. To our knowledge, no English-language reports from Japan have been published to date. Thus, there may be limited awareness among Japanese urologists regarding specific techniques and practical tips for the effective use of FloSeal®. This case report aims to provide a detailed, step-by-step description of our institution’s reproducible FloSeal®-assisted RAPN technique, which uses a flexible feeding tube after inner-layer renorrhaphy to achieve adequate parenchymal hemostasis.

## Case presentation

Case 1

**Figure 1:**
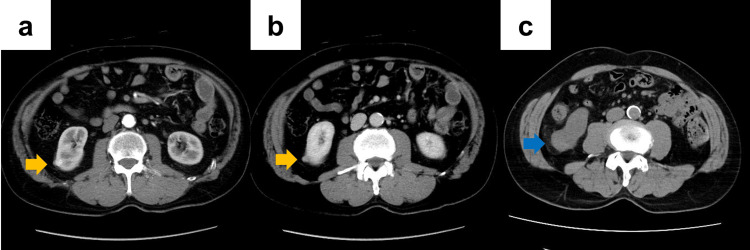
Computed tomography findings in Case 1 (a, b) Initial dynamic contrast-enhanced CT demonstrates a 10-mm exophytic tumor located at the lateral portion of the lower pole of the right kidney (a: early phase; b: portal venous phase). (c) After seven years of active surveillance, the tumor has increased in size to 30 mm. Arrows indicate the renal tumor.

A 67-year-old man (body mass index, 24.2 kg/m²) had a medical history of surgery for nasopharyngeal carcinoma and papillary thyroid carcinoma, in addition to hypertension and dyslipidemia. A 10-mm right renal mass was incidentally identified on computed tomography (CT) performed for postoperative surveillance of nasopharyngeal carcinoma seven years earlier, and the patient was referred to our hospital for further evaluation. Repeated dynamic contrast-enhanced CT revealed a right renal tumor with intense enhancement in the arterial phase and washout in the portal venous phase (Figures 1a, 1b), findings consistent with clear cell renal cell carcinoma (RCC). Because the patient initially preferred active surveillance, the lesion was monitored with periodic imaging. However, the mass was gradually enlarged and eventually reached a maximum diameter of 30 mm (Figure 1c). The lesion was diagnosed as right RCC (clinical stage T1aN0M0, RENAL nephrometry score 4x), and RAPN via a transperitoneal approach was planned. The preoperative estimated glomerular filtration rate (eGFR) was 50.8 mL/min/1.73 m^2^.

Surgical technique

**Figure 2:**
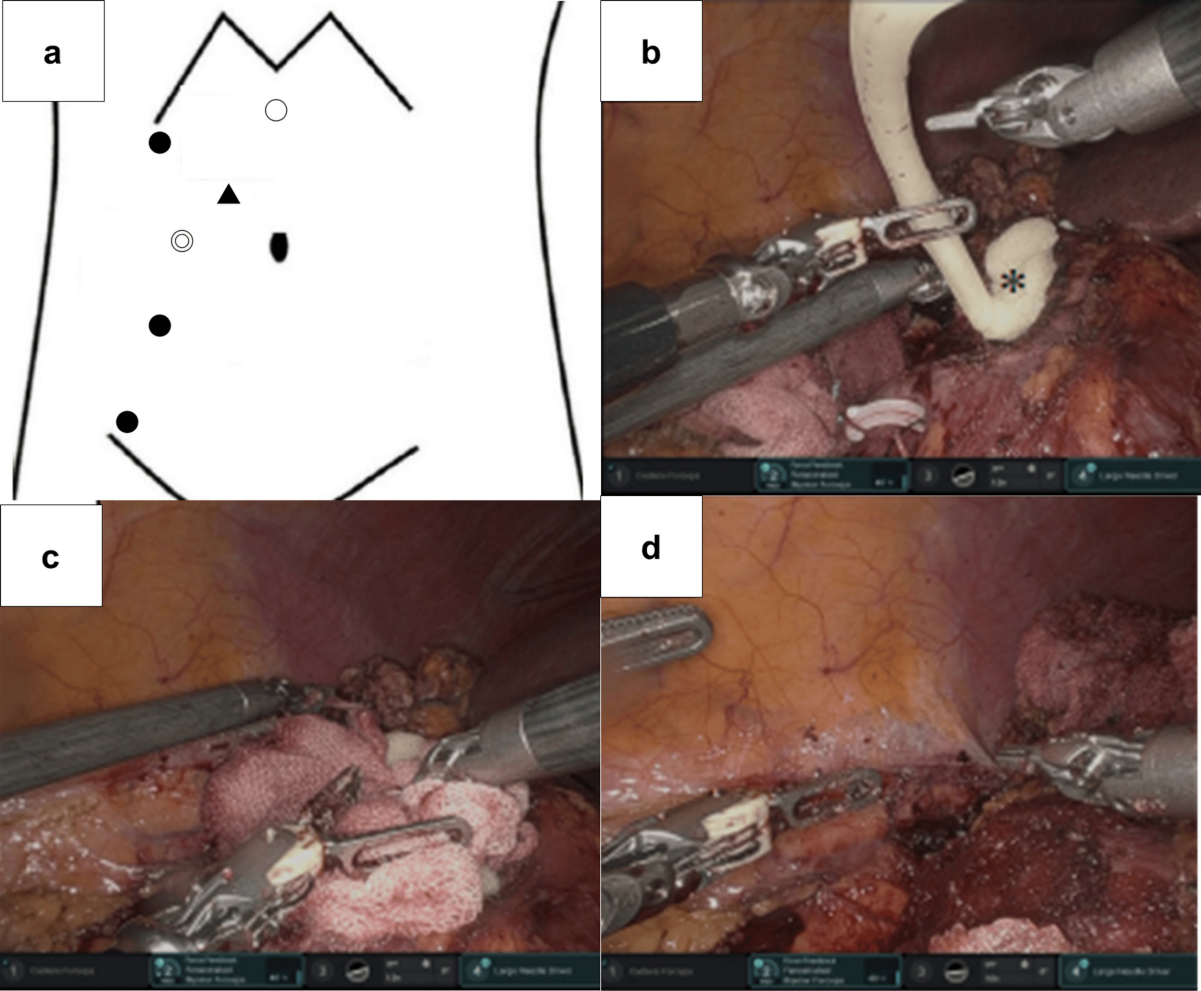
Port placement and intraoperative application of FloSeal® in Case 1 Port placement and intraoperative application of FloSeal® in Case 1 (a) Port placement for RAPN via a transperitoneal approach (◎: camera port; ●: da Vinci ports; ▲: assistant port; ○: port for liver retraction). (b) Application of FloSeal® to the tumor bed using a flexible feeding tube, demonstrating targeted delivery of the flowable hemostatic matrix. (c) Gentle compression of the FloSeal® layer with saline-moistened surgical gauze to compact the matrix and promote clot formation. (d) Complete parenchymal hemostasis achieved after removal of the gauze, without the need for additional cortical suturing. (*) indicates FloSeal®.

RAPN via a transperitoneal approach was performed in the standard fashion using the da Vinci 5® system (Intuitive Surgical, Sunnyvale, CA, USA). The port placement is illustrated in Figure 2a. Following the clamping of the right renal artery, the lower-pole tumor was excised according to the standard procedure. The tumor bed was carefully coagulated using monopolar coagulation with monopolar scissors and bipolar coagulation with fenestrated bipolar forceps, with the objective of achieving hemostasis to the greatest extent possible. Subsequently, an inner layer renorrhaphy of the resection bed was performed using a 3-0 Stratafix™ suture (Ethicon, Cincinnati, OH, USA), followed by unclamping of the renal artery. The warm ischemia time (WIT) was 18 minutes.

Subsequent to the unclamping procedure, a 14-Fr feeding tube (Safeed®, Create Medic, Tokyo, Japan) was inserted through the 12-mm assistant port (ENDOPATH XCEL™ bladeless trocar, Ethicon Endo-Surgery, Cincinnati, OH, USA). As the distal tip of the Safeed® feeding tube has lateral side holes, the tip was trimmed prior to utilization. Thereafter, FloSeal® was applied in a liberal manner to the tumor bed, resulting in the formation of a substantial layer over the resection surface (Figure 2b). The favorable flexibility of the feeding tube and the high maneuverability of the robotic instruments enabled the precise delivery of FloSeal® to the intended sites. A gauze pad moistened with a small amount of normal saline was then gently pressed against the tumor bed to compact the FloSeal® layer (Figure 2c). Two minutes later, the gauze was removed, revealing a well-formed fibrin clot within the gelatin-thrombin matrix with complete hemostasis (Figure 2d).

Peri- and postoperative results

RAPN was successfully completed without conversion to conventional laparoscopic PN or open surgery. The console time and estimated blood loss were 79 minutes and 5 mL, respectively. The postoperative course was uneventful; the drain was removed on postoperative day (POD) 2, and the patient was discharged on POD 5.

**Figure 3:**
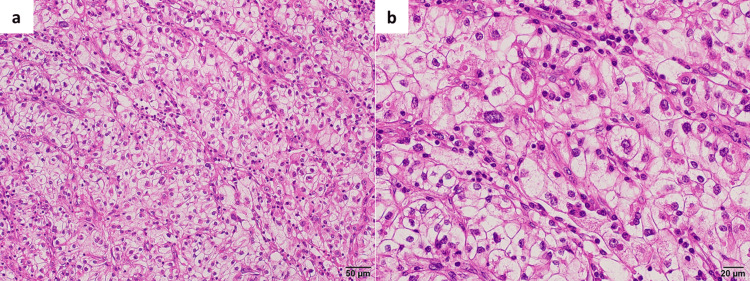
Histopathological findings in Case 1 (a) Tumor cells with optically clear cytoplasm proliferate in a nested architecture. (b) A higher-power view demonstrates conspicuous nucleoli that are readily appreciable even at low magnification, consistent with WHO/ISUP nuclear grade 3. No sarcomatoid or rhabdoid differentiation is identified, and no coagulative tumor necrosis is present. Hematoxylin and eosin staining. Scale bars: 50 μm (a) and 20 μm (b).

Histopathological examination revealed clear cell RCC, WHO/ISUP grade 3, without venous invasion (Figure 3), and the tumor was staged as pT1a. The surgical margin was negative. At one month postoperatively, the eGFR was 49.8 mL/min/1.73 m².

Case 2

**Figure 4:**
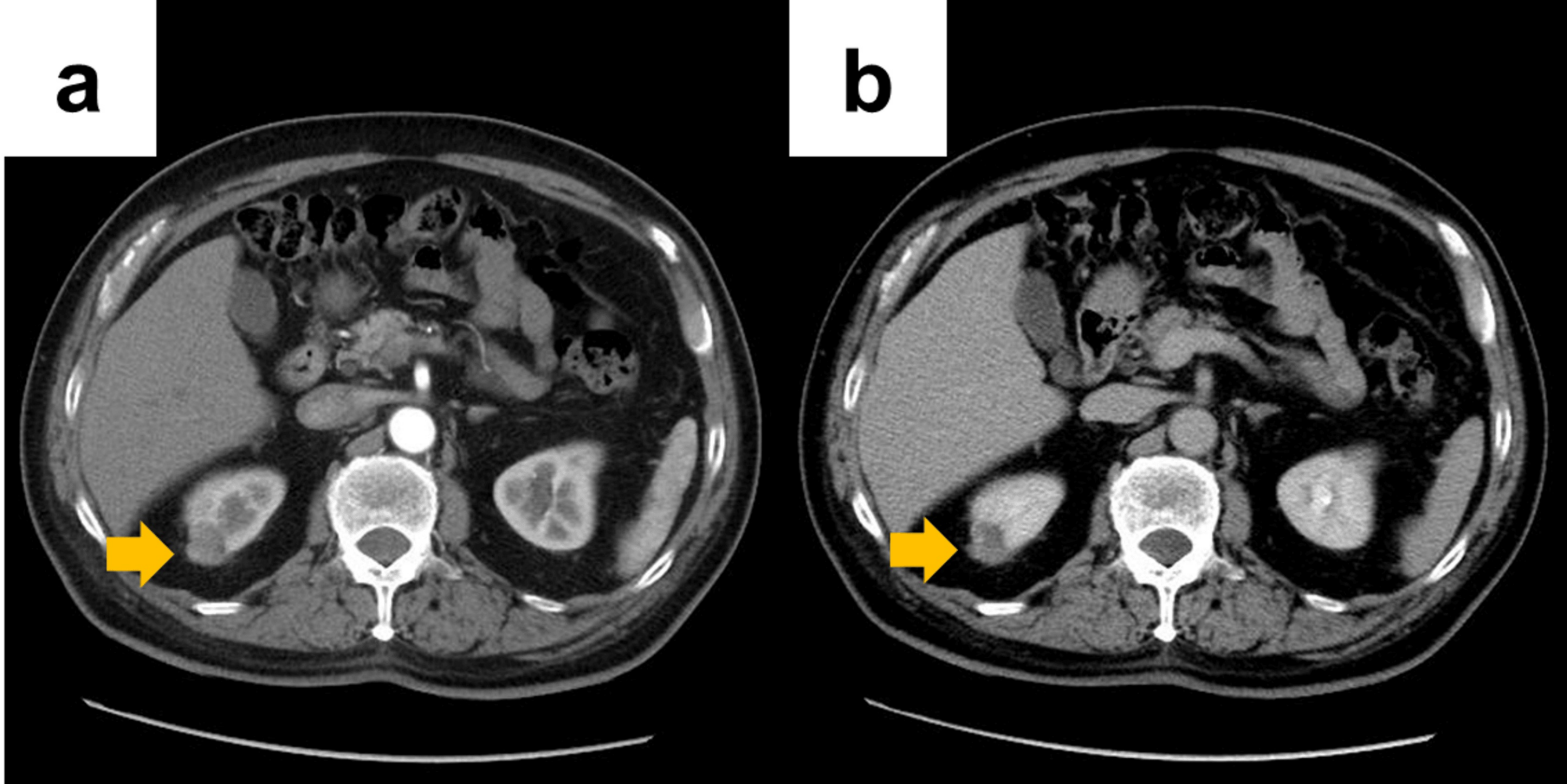
Computed tomography findings in Case 2 (a, b) Dynamic contrast-enhanced CT shows a 22-mm tumor at the lateral portion of the upper pole of the right kidney (a: early phase; b: excretory phase). Arrows indicate the renal tumor.

A 74-year-old male patient (body mass index, 26.4 kg/m²) with a medical history of lung cancer surgery, diabetes mellitus, and benign prostatic hyperplasia was incidentally found to have a 10 mm right renal mass on computed tomography performed at a local clinic six years earlier. The lesion was monitored with periodic imaging, during which it gradually increased in size. Consequently, the patient was referred to our institution for further evaluation and treatment. Contrast-enhanced dynamic CT demonstrated a 22 mm mass in the upper pole of the right kidney with progressively increasing enhancement (Figures 4a, 4b). The lesion was diagnosed as right renal cell carcinoma (clinical stage T1aN0M0, RENAL nephrometry score 4x) and RAPN via a retroperitoneal approach was planned. The preoperative eGFR was 66.5 mL/min/1.73 m^2^.

Surgical technique

**Figure 5:**
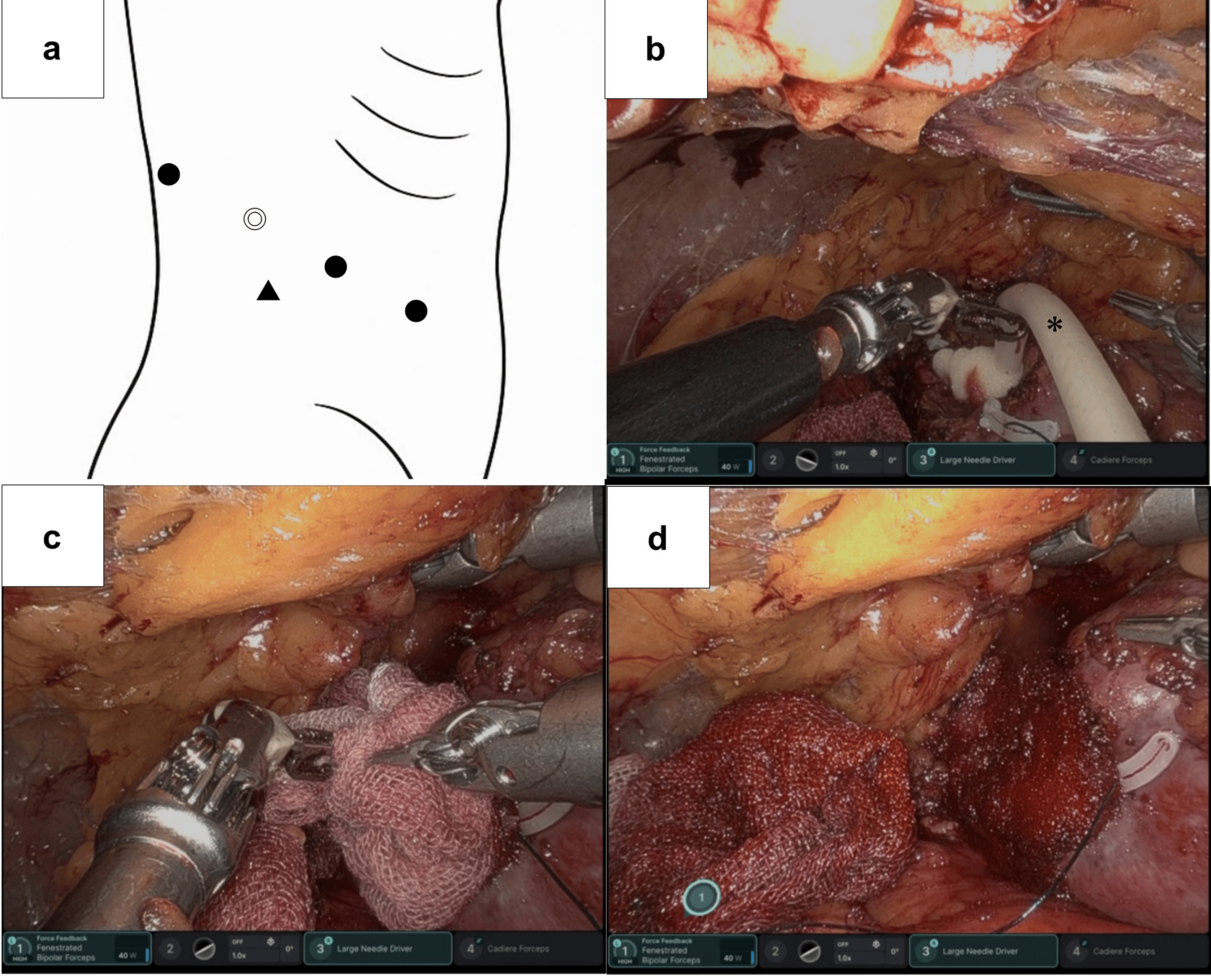
Port placement and intraoperative application of FloSeal® in Case 2 Port placement and intraoperative application of FloSeal® in Case 2 (a) Port placement for RAPN via a retroperitoneal approach (◎: camera port; ●: da Vinci ports; ▲: assistant port). (b) Application of FloSeal® to the tumor bed using a flexible feeding tube, illustrating targeted delivery of the flowable hemostatic matrix. (c) Gentle compression of FloSeal® layer with saline-moistened surgical gauze to compact the matrix and promote clot formation. (d) Complete parenchymal hemostasis confirmed after gauze removal, without the need for additional cortical suturing. (*) indicates FloSeal®.

RAPN via a retroperitoneal approach was carried out in accordance with standard surgical protocols using the da Vinci 5® surgical system. The port placement is illustrated in Figure 5a. Following the clamping of the right renal artery, the upper-pole tumor was excised in accordance with standard technique. As in Case 1, meticulous coagulation of the tumor bed was carried out to achieve maximal hemostasis. This was followed by inner layer renorrhaphy and subsequent unclamping of the renal artery. WIT was 18 minutes. Following the unclamping procedure, FloSeal® was applied to the resection bed using the same technique employed in Case 1, thereby facilitating the reliable and efficient achievement of complete hemostasis (Figures 5b-5d).

Peri- and postoperative results

RAPN was successfully completed without conversion to conventional laparoscopic PN or open surgery. The console time and estimated blood loss were 93 minutes and 10 mL, respectively. The postoperative course was uneventful; the drain was removed on POD 2, and the patient was discharged on POD 5.

**Figure 6:**
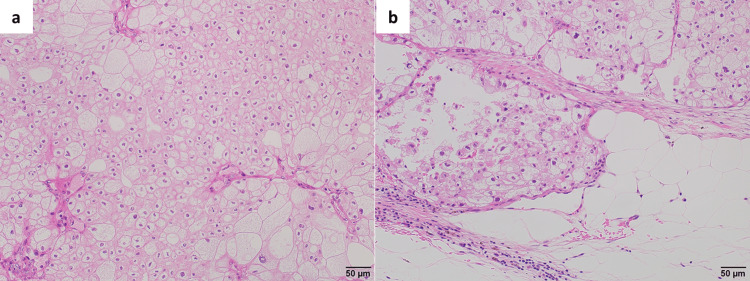
Histopathological findings in Case 2 (a) The tumor is composed of large–sized cells with hyperchromatic and irregular-shaped nuclei, abundant pale cytoplasm and prominent cell borders, arranged in a solid pattern and surrounded by incomplete fibrovascular septa. No sarcomatoid or rhabdoid differentiation and no coagulative tumor necrosis are identified. (b) Focal invasion into the adjacent perirenal adipose tissue is present. Hematoxylin and eosin staining. Scale bars: 50 μm (a) and 50 μm (b).

Histopathological examination revealed chromophobe RCC without venous invasion, and pathological stage pT3a due to perinephric fat invasion (Figure 6). The surgical margin was negative. At one month postoperatively, eGFR was 67.4 mL/min/1.73 m² in Case 2.

## Discussion

In this two-case technical report, we describe a simple technique using a gelatin-thrombin matrix sealant (FloSeal®) delivered through a flexible feeding tube as an adjunct to inner-layer renorrhaphy during RAPN. Both cases were completed safely without conversion or bleeding-related complications, and each achieved the commonly used trifecta for PN: negative surgical margins, WIT < 25 minutes, and absence of perioperative complications [7]. These findings suggest that the present approach can achieve stable parenchymal hemostasis without extensive cortical suturing. To our knowledge, this is the first English-language report from Japan to detail a FloSeal®-assisted RAPN technique using a flexible feeding tube.

Although both tumors in this series were classified as low complexity, we intentionally selected these relatively simple exophytic lesions as our initial experience with FloSeal®-assisted RAPN. Introducing the technique under low-risk anatomical conditions allowed us to standardize each procedural step, ensure reproducibility, and evaluate feasibility and safety in a stepwise, controlled manner. Previous studies have demonstrated that larger tumor size, higher RENAL nephrometry scores, central or completely endophytic masses, proximity to the collecting system or renal sinus, and limited surgeon experience are associated with prolonged WIT, technically demanding renorrhaphy, and lower rates of trifecta achievement [8-10]. On this basis, the present technique may be particularly useful in higher-complexity cases in which prolonged WIT and difficult renorrhaphy are expected, as well as for surgeons early in their learning curve who must achieve secure hemostasis and adequate suturing within a limited ischemic window. Larger cohorts that include more complex renal tumors are needed to validate this hypothesis.

FloSeal® adheres well even to irregular tissue surfaces and maintains its effectiveness in the presence of active bleeding. This facilitates hemostasis in confined surgical fields where suturing is challenging and in hard-to-reach areas. Owing to its efficacy and ease of use, FloSeal® is widely used across various surgical specialties, including cardiovascular surgery, neurosurgery, otolaryngology, gynecology, and general surgery [11-13]. It consistently controls diffuse oozing and localized arterial bleeding, even when suturing or electrocoagulation is insufficient. Nevertheless, its adoption in the field of urology in Japan is limited.

The usefulness of FloSeal® in PN has been reported. In open, laparoscopic, and robotic PN, gelatin-thrombin matrix sealants, including FloSeal®, achieved rapid control of parenchymal bleeding with a low incidence of bleeding-related complications and without deterioration of renal function or an increase in perioperative complications [1,6,14-16]. Although the choice between hemostasis based primarily on topical agents and that based on parenchymal suturing requires further investigation, parenchymal sutures have been implicated as a potential cause of postoperative vascular complications such as arteriovenous fistulas and pseudoaneurysm formation [17]. Topical agents, such as FloSeal®, can provide hemostasis without excessive tissue compression or needle-induced vascular injury; thus, they may help preserve renal function and reduce the risk of vascular complications.

From a practical standpoint, potential indications for considering FloSeal® during RAPN include: (1) diffuse parenchymal oozing that persists despite meticulous bipolar and monopolar coagulation, (2) deep or confined resection beds, particularly when close to the renal sinus or collecting system, and (3) fragile or thinned renal parenchyma in which extensive cortical suturing may risk tissue tearing or prove technically challenging.

 In Japan, similar concepts have led surgeons to adopt strategies such as meticulous soft coagulation of the resection bed or the application of sheet-type hemostatic materials. Compared with these approaches, the use of a gelatin-thrombin matrix sealant is technically simple, can be completed in a short time, and offers excellent reproducibility. In the present cases, we used a low-cost flexible feeding tube to deliver FloSeal® into the operative field. The favorable flexibility of the tube combined with the high maneuverability of the robotic instruments allowed precise and straightforward application of the sealant to the intended locations on the resection bed.

## Conclusions

Our cases suggest that flowable gelatin-thrombin matrix sealants can serve as a reliable adjunct to suture-based hemostasis in PN. Incorporating these agents into a structured, stepwise hemostatic strategy may help preserve nephron function while minimizing the risk of bleeding and vascular complications. Future prospective and comparative studies are warranted to investigate optimal indications, refine technical nuances, and further define the role of these sealants in contemporary nephron-sparing surgery.
